# Diagnostic value of multiple ultrasound diagnostic techniques for axillary lymph node metastases in breast cancer: A systematic analysis and network meta-analysis

**DOI:** 10.3389/fonc.2022.1043185

**Published:** 2023-01-06

**Authors:** Jun Li, Si-Rui Wang, Qiao-Li Li, Tong Zhu, Pei-Shan Zhu, Ming Chen, Xin-Wu Cui

**Affiliations:** ^1^ Department of Medical Ultrasound, the First Affiliated Hospital of Medical College, Shihezi University, Xinjiang, China; ^2^ NHC Key Laboratory of Prevention and Treatment of Central Asia High Incidence Diseases (First Affiliated Hospital, School of Medicine, Shihezi University), Shihezi, Xinjiang, China; ^3^ School of Medicine, Shihezi University, Shihezi, China; ^4^ Department of Medical Ultrasound, Tongji Hospital, Tongji Medical College, Huazhong University of Science and Technology, Wuhan, China

**Keywords:** ultrasound, ultrasound elastography, contrast-enhanced ultrasound, breast cancer, lymph nodes metastasis, network meta-analysis

## Abstract

**Background:**

Early diagnosis of axillary lymph node metastasis is very important for the recurrence and prognosis of breast cancer. Currently, Lymph node biopsy is one of the important methods to detect lymph node metastasis in breast cancer, however, its invasiveness might bring complications to patients. Therefore, this study investigated the diagnostic performance of multiple ultrasound diagnostic methods for axillary lymph node metastasis of breast cancer.

**Materials and methods:**

In this study, we searched PubMed, Web of Science, CNKI and Wan Fang databases, conducted Bayesian network meta-analysis (NMA) on the studies that met the inclusion criteria, and evaluated the consistency of five different ultrasound imaging techniques in axillary lymph node metastasis of breast cancer. Funnel graph was used to evaluate whether it had publication bias. The diagnostic performance of each ultrasound imaging method was ranked using SUCRA

**Results:**

A total of 22 papers were included, US+CEUS showed the highest SUCRA values in terms of sensitivity (SEN) (0.874), specificity (SPE) (0.911), positive predictive value (PPV) (0.972), negative predictive value (NPV) (0.872) and accuracy (ACC) (0.990).

**Conclusion:**

In axillary lymph node metastasis of breast cancer, the US+CEUS combined diagnostic method showed the highest SUCRA value among the five ultrasound diagnostic methods. This study provides a theoretical basis for preoperative noninvasive evaluation of axillary lymph node metastases in breast cancer patients and clinical treatment decisions.

**Systematic review registration:**

https://www.crd.york.ac.uk/PROSPERO/, identifier CRD42022351977.

## 1 Introduction

Breast Cancer is the most common malignancy tumor in women worldwide, and its incidence is much higher than other cancers (1), it ranks first in incidence and second in mortality among female malignant tumors (2). The occurrence of axillary lymph nodes metastasis is a key factor affecting the recurrence and prognosis of breast cancer. In order to avoid the spread of cancer cells through lymph nodes, axillary lymph node dissection is often performed in breast cancer patients. Although this method can effectively inhibit the recurrence of breast cancer and improve the prognosis, it may cause a series of complications, such as lymph node edema, Cellulitis, etc. Currently, the axillary staging and treatment of early breast cancer has changed from complete axillary lymph node dissection to sentinel lymph node biopsy (SLNB), which has a higher accuracy rate and a lower rate of postoperative complications (3). However, as an invasive procedure, SLNB may still lead to postoperative complications such as subcutaneous effusion, nerve injury, and restriction of shoulder joint movement, and the incidence of SLNB is 7.1% (4). Therefore, an accurate assessment of the extent of axillary lymph node involvement by non-invasive methods before surgery can minimize the incidence of postoperative complications caused by invasive methods. In non-invasive diagnosis, the sensitivity (SEN) of axillary lymph node palpation is only 33% to 68% (5), computer tomography (CT), positron emission tomography (PET) and other diagnosis methods (6) have the disadvantages of high price, radiation, etc., and do not show the obvious correlation in the evaluation of axillary lymph node metastasis in breast cancer (7, 8).

As one of the main detection methods of non-invasive imaging, ultrasound (US) has the advantages of no radiation, economy, convenience, and real-time imaging, and has become a common imaging method for the diagnosis of axillary lymph node metastasis in breast cancer. However, some studies have shown that 2D ultrasound has low SEN and specificity(SPE) in detecting benign and malignant lymph nodes due to its poor imaging of deep axillary lymph nodes and inability to show typical morphological changes (9). Ultrasound elastography (UE), contrast-enhanced ultrasound (CEUS), and other techniques may allow better differentiation between benign and malignant masses (10, 11). Studies have shown that elastography has high diagnostic performance in distinguishing benign from metastatic LNs, however, Park et al (12) showed that elastography did not have a significant advantage in evaluating metastatic lymph nodes. Tsai et al (13) found that US+UE showed higher SEN and SPE than US and UE alone. With the continuous progress of ultrasound technology, CEUS is widely used in clinical practice, and has higher SEN and SPE for lymph node metastasis, so that accuracy of diagnosing axillary lymph node metastasis in breast cancer is better improved.

The diagnostic performance of ultrasound diagnostic techniques for breast Cancer axillary lymph nodes is still controversial, and the results obtained by different clinical trials are also different. Therefore, we comprehensively analyze the diagnostic performance of US, UE, CEUS, US+UE, and US+CEUS.

This study conducted an NMA of the diagnostic performance of US, UE, CEUS, US+UE, and US+CEUS using two or more published studies of ultrasound imaging methods, comparison of different ultrasound imaging techniques for detection of SEN, SPE, positive predictive value (PPV), negative predictive value (NPV), accuracy (ACC) in axillary lymph node metastases. Helping clinicians find more accurate methods for diagnosing axillary lymph node metastases in breast cancer thereby improving patient outcomes.

## 2 Method

### 2.1 Retrieval strategy

We searched for relevant studies published in Chinese National Knowledge Infrastructure, PubMed, Web of Science, and Wan Fang before July 2022. Using “lymph node”, “Lymphatic Metastasis”, Elasticity imaging Techniques”, “Ultrasonography”, “Breast” cancer”, “ Contrast Ultrasound “ and other keywords were searched (**Table 1**). The included references were also screened to ensure that all included references met the inclusion and exclusion criteria.

**Table 1:** Search strategy.

No.	Retrieval type
#1	lymph node【Mesh】
#2	Neoplasm Staging【Mesh】
#3	Staging, Neoplasm【Title/Abstract】
#4	Tumor Staging【Title/Abstract】
#5	TNM Staging System【Title/Abstract】
#6	TNM Classifications【Title/Abstract】
#7	Preoperative Staging【Title/Abstract】
#8	Lymphatic Metastasis【Mesh】
#9	Lymphatic Metastases【Title/Abstract】
#10	Lymph Node Metastasis【Title/Abstract】
#11	Lymph Nodes Metastasis【Title/Abstract】
#12	Metastasis, Lymph Node【Title/Abstract】
#13	Axilla 【Title/Abstract】
#14	#1OR #2OR #3OR #4OR #5OR #6OR #7OR #8OR #9OR #10OR #11OR #12OR #13
#15	Ultrasound Contrast【Title/Abstract】
#16	Elasticity Imaging Techniques【Mesh】
#17	Elastography【Title/Abstract】
#18	Elastogram【Title/Abstract】
#19	B-mode【Title/Abstract】
#20	Ultrasonography【Mesh】
#21	Diagnostic Ultrasound【Title/Abstract】
#22	Ultrasound Imaging【Title/Abstract】
#23	Ultrasonic Imaging【Title/Abstract】
#24	Ultrasonic Diagnosis【Title/Abstract】
#25	Ultrasound Diagnosis【Title/Abstract】
#26	#15OR #16OR #17OR #18OR #19OR #20OR #21OR#22 OR#23 OR #24OR #25
#27	Breast Neoplasms【Mesh】
#28	Breast Tumors【Title/Abstract】
#29	Mammary Cancer【Title/Abstract】
#30	Breast Malignant Neoplasm【Title/Abstract】
#31	Breast Malignant Tumors【Title/Abstract】
#32	Human Mammary Carcinoma【Title/Abstract】
#33	Breast Carcinoma【Title/Abstract】
#34	Breast Cancer【Title/Abstract】
#35	#27 OR#28 OR#29OR #30 OR#31OR#32#33 OR#34
#36	#14 AND#26 AND#35

### 2.2 Research screening

The relevant inclusion criteria are as follows: 1) Population: patients with pathologically proven breast cancer with axillary lymph node metastasis; 2) Diagnosis method: including two or more ultrasound imaging methods; 3) Study result should include calculable indicators such as true-positive (TP), false-positive (FP), true-negative (TN), false-negative (FN) of describe the diagnostic performance of the study; 4) Type of study: diagnostic trial.

The relevant exclusion criteria include the following aspects: (1) The study population is non-human studies or studies with axillary lymph node metastases of breast cancer without pathological confirmation; (2) the diagnostic performance indicators in the studies are incomplete; (3) Editorials, reviews, case reports, meeting minutes, guidelines, etc.

The titles and abstracts of retrieved articles were read by two authors, respectively. studies that do not meet the inclusion criteria will be excluded according to the inclusion and exclusion criteria established in this study.

### 2.3 Data extraction

Data extraction was performed on the originally included studies and was independently extracted by two investigators. The extracted data included: 1) The first author; 2) Research publication time; 3) Country of the first author; 4) The mean of the patient’s age; 5) Diagnostic method; 6) Sample size; 7) The results of the study were TP, FP, TN, FN.

### 2.4 Statistical analysis

This meta-analysis has been registered on the PROSPERO website with registration number CRD42022336701.We divided the different ultrasound diagnostic methods in the included study into five groups, namely US, UE, CEUS, US+UE, and US+CEUS, and used NMA to analyze the diagnostic performance of the five groups in the diagnosis of axillary lymph node metastasis in breast cancer. According to the PRISMA NMA list, Stata’s(version-15.1) -Markov chain Monte Carlo model was used. The NMA was aggregated and analyzed in a Bayes-based framework, and the five groups of data were compared directly and indirectly. The diagnostic performance of each diagnostic method was judged by analyzing its SEN, SPE, PPV, NPV, and ACC indicators, and using the P value or I^2^ to evaluate heterogeneity. P value <0.05 or I^2^>90% indicates that the heterogeneity was large.

We also use the nodal method to evaluate the inconsistency in NMA, using the surface under the cumulative ranking curve (SUCRA) to calculate the probability of each imaging mode. The value of SUCRA is between 0 and 1(0≤SUCRA ≤ 1), when SUCRA is 1, it indicates that the intervention is absolutely effective, and when SUCRA is 0, it indicates that the intervention is absolutely ineffective. According to the value of SUCRA, the pros and cons of the diagnostic methods can be sorted, so as to screen out the most effective diagnostic methods.

**Figure 1:**
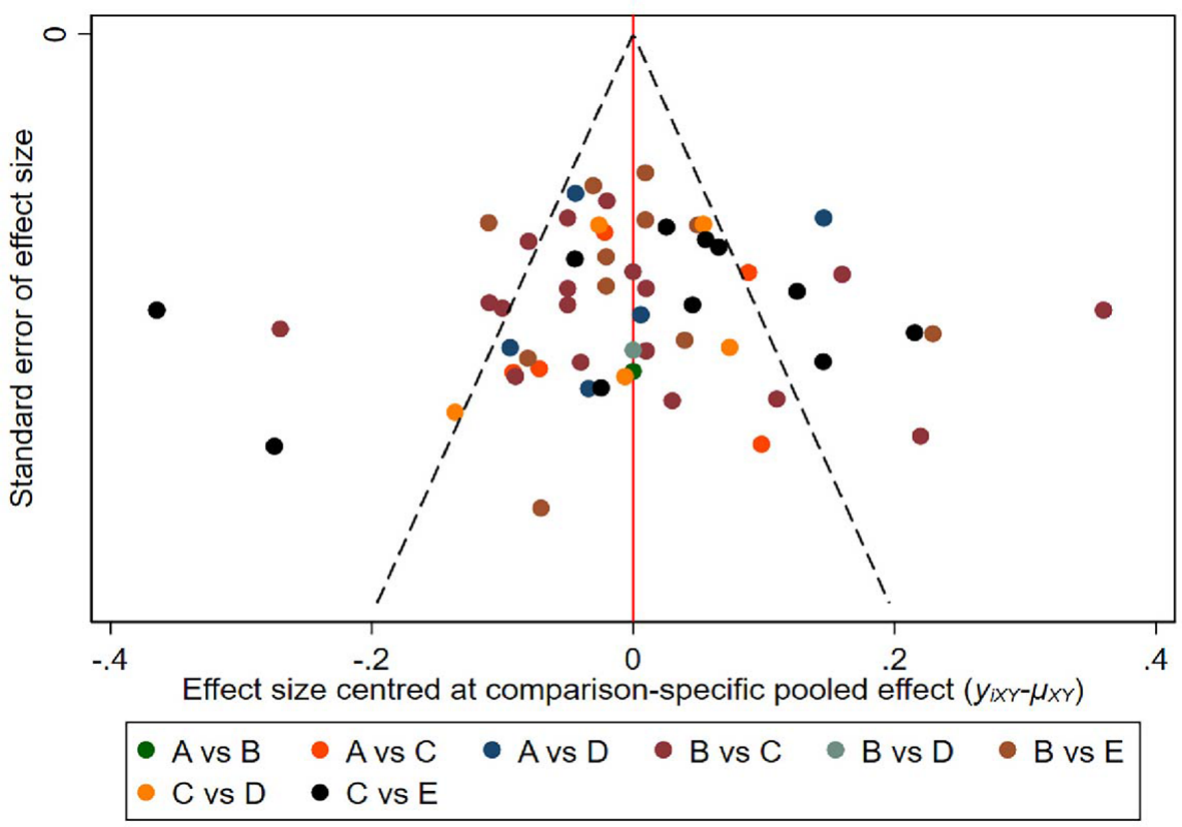
Funnel plot on publication bias (A=CEUS, B=UE, C=US, D=US+CEUS, E=US+UE).

This study used funnel plots to detect possible publication bias, and the results showed that the distribution of funnel plots was roughly symmetric, suggesting that there was no publication bias or other bias in the study (**Figure 1**).

## 3 Results

### 3.1 Literature selection

This study found 8072 studies from the database based on keywords, of which 1999 articles were extracted from PubMed, 3502 articles were extracted from Web of Science,1214 articles were extracted from Wan Fang, and 1357 articles were extracted from CNKI. A total of 8050 studies that did not meet the inclusion criteria were excluded from this study, and 22 studies were finally included (3, 10, 12–31) (**Table 2**). We included published studies using two or more ultrasound imaging methods, and analyzed and evaluated the extracted diagnostic indicators.

**Table 2:** Flow diagram of literature selection.

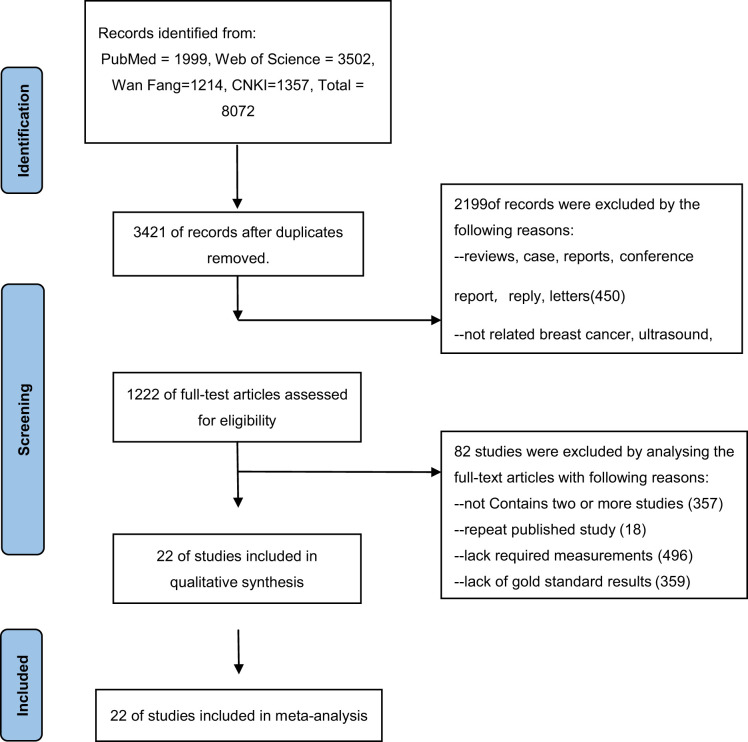

### 3.2 Study characteristics

**Figure 2:**
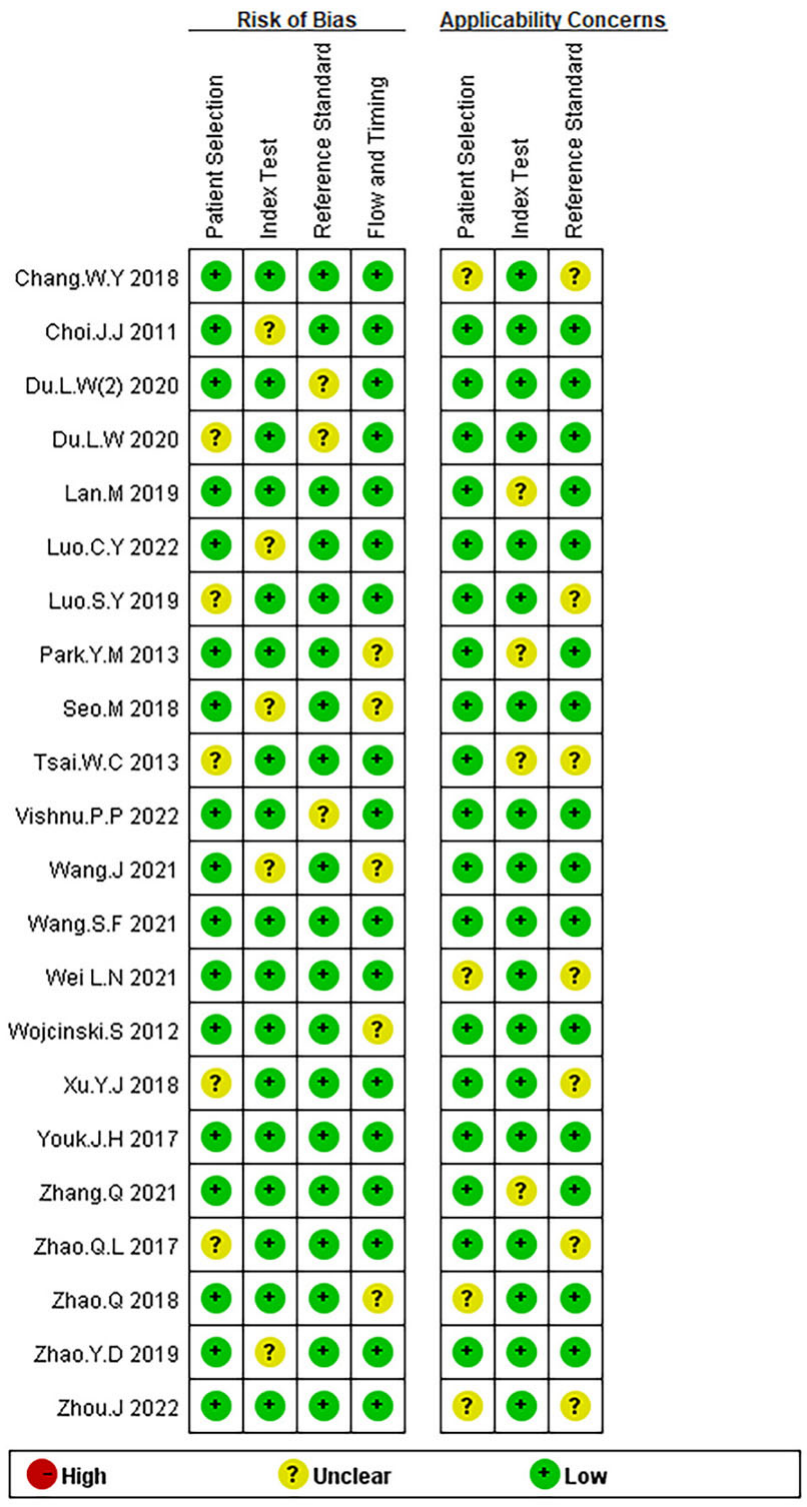
Bias risk of the included studies (QUADAS 2 criteria).

A total of 7776 patients (range, 42-313) were included in 22 studies (3, 10, 12–31), all of whom were pathologically confirmed to have lymph node metastases of breast cancer. Among these studies, there were 2 retrospective studies and 20 randomized controlled studies. There were many studies on the US, UE, and US+UE in the included literature, among which 18 studies compared US vs UE,11 studies compared US vs US+UE, 11 studies compared UE vs US+UE, 5 studies compared US vs CEUS. Five studies compared US vs US+CEUS (**Table 3**). The quality assessment of the literature was based on QUADAS-2 scale to evaluate 22 studies from four aspects: Patient Selection, Reference Standard, Index Test, and Flow Timing. The results show that the overall quality of the included studies was relatively satisfactory (**Figure 2**). Among the 22 articles, 5 had an unclear risk of bias in the Index Test, which may be due to the differences in the operators performing the tests and their experience levels.

**Table 3:** Overview of characteristics of all included studies.

Author	Year	country	Design	Age, Mean (Range)	Patient number	Gold standard	Diagnostic method		
ZHAO Q.	2018	China	Pro	53.1 (31-77)	313	Pathologic	①	②	
Choi J. J.	2011	Korea	RCT	53 (27-81)	62	Pathologic	①	②	④
Zhou J.	2022	China	RCT	/	160	Pathologic	①	②	
Wojcinski S.	2012	Germany	RCT	/	180	Pathologic	①	②	④
TSAI W. C.	2013	China (Taiwan)	RCT	51 (20-84)	89	Pathologic	①	②	④
Chang W. Y.	2018	China	RCT	55.3 (21-85)	140	Pathologic	①	②	④
Xu Y. J.	2018	China	RCT	/	97	Pathologic	①	②	④
Park Y. M.	2013	American	RCT	55 (33-99)	101	Pathologic	①	②	
Zhao Q. L.	2017	China	RCT	52.47 (27-79)	78	Pathologic	①	②	④
Luo C. Y.	2022	China	RCT	49.5 (41-58)	114	Pathologic	①	②	④
Lan M.	2019	China	RCT	50.5 (22-78)	107	Pathologic	①	②	
Vishnu P. P.	2022	India	RCT	46.3 (34-58)	54	Pathologic	①	②	④
Wei L. N.	2021	Malaya	RCT	58 (33-82)	107	Pathologic	①	②	④
Wang J.	2021	China	RCT	42.4 (35-78)	85	Pathologic	①	②	④
Seo M.	2018	Korea	RCT	54.7 (33-80)	66	Pathologic	①	②	
Youk J. H.	2017	Korea	RCT	/	130	Pathologic	①	②	
Luo S. Y.	2019	China	RCT	46.68 (27-69)	158	Pathologic	①	②	④
Du L. W.	2020	China	RCT	49.4 (24-84)	234	Pathologic	①	③	⑤
Zhang Q.	2021	China	RCT	50.5 (32-77)	120	Pathologic	①	②	③
Du L. W.	2020	China	RCT	49.4 (24-85)	234	Pathologic	①	③	⑤
Zhao Y. D.	2019	China	RCT	44.4 (28-59)	42	Pathologic	①	③	⑤
Wang S. F.	2021	China	RCT	48.4 (25-70)	120	Pathologic	①	③	⑤

①:Ultrasound; ②:Ultrasonic elasticity; ③:Contrast-enhance ultrasound; ④:Ultrasound+ Ultrasonic elasticity; ⑤:Ultrasound+ Contrast-enhance ultrasound.

### 3.3 Network meta-analysis

**Figure 3:**
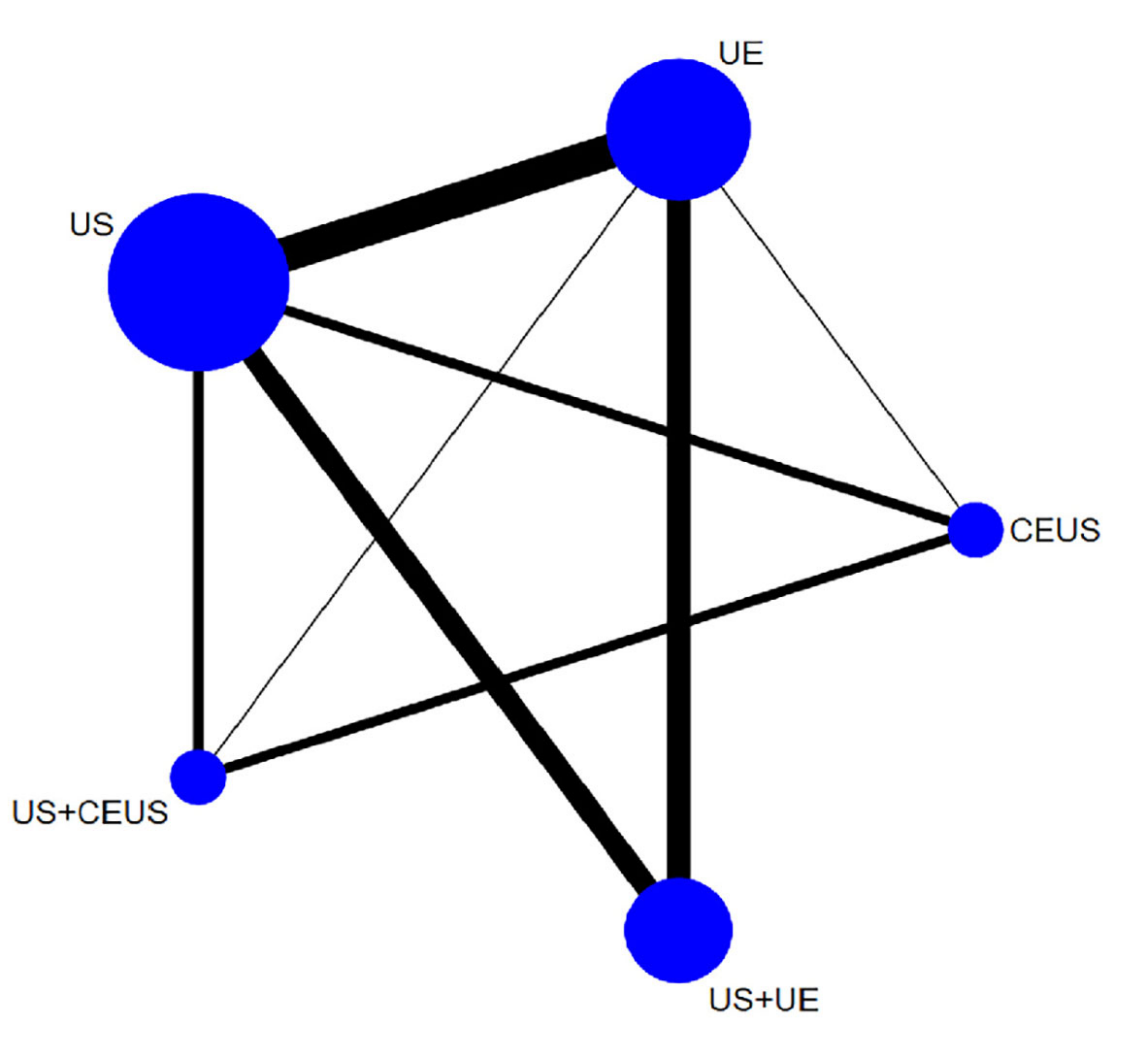
Network Mate-Analysis Figure.

The Network evidence diagram was shown in **Figure 3**. In this study, the consistency of direct comparison and indirect comparison of the included studies was analyzed, and the results showed that all studies were P > 0.05, indicating that the studies had good consistency.

#### 3.3.1 SEN

**Figure 4:**
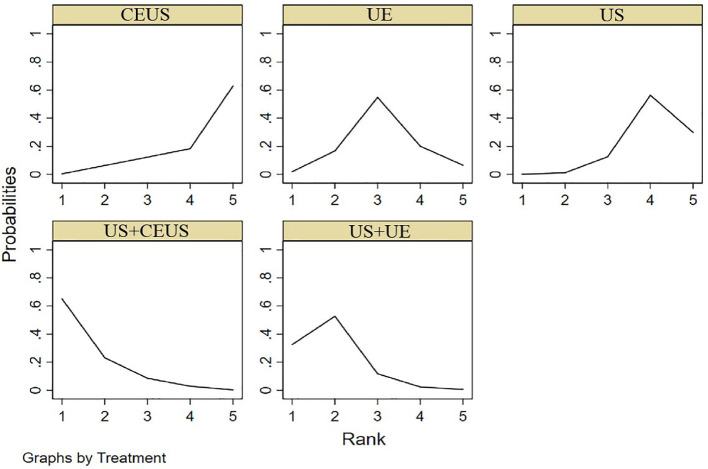
SUCRA plot for SEN.

NMA showed that US+CEUS [MD=0.15, 95%CI (0.02, 0.28)] was superior to the control group (CEUS) in diagnosing SEN in axillary lymph node metastasis of breast cancer (**Table 4A**). US+CEUS ranked first in SEN for axillary lymph node metastasis of breast cancer in different methods(SUCRA: 87.4% as shown in **Table 5**) (**Figure 4**).

**Table 4:** League table on five kinds of diagnostic efficacy.

US+CEUS	US+UE	UE	US	CEUS
**A. League table on SEN**				
US+CEUS	-0.04 (-0.19,0.12)	-0.08 (-0.23,0.06)	-0.12 (-0.25,0.00)	-0.15 (-0.28, -0.02)
0.04 (-0.12,0.19)	US+UE	-0.05 (-0.13,0.04)	-0.09 (-0.18, -0.00)	-0.11 (-0.27,0.04)
0.08 (-0.06,0.23)	0.05 (-0.04,0.13)	UE	-0.04 (-0.11,0.03)	-0.07 (-0.21,0.08)
0.12 (-0.00,0.25)	0.09 (0.00,0.18)	0.04 (-0.03,0.11)	US	-0.02 (-0.15,0.11)
0.15 (0.02,0.28)	0.11 (-0.04,0.27)	0.07 (-0.08,0.21)	0.02 (-0.11,0.15)	CEUS
**B. League table on SPE**				
US+CEUS	CEUS	US+UE	UE	US
US+CEUS	-0.04 (-0.18,0.10)	-0.14 (-0.30,0.02)	-0.16 (-0.31, -0.01)	-0.21 (-0.35, -0.07)
0.04 (-0.10,0.18)	CEUS	-0.10 (-0.27,0.06)	-0.12 (-0.28,0.03)	-0.17 (-0.31, -0.03)
0.14 (-0.02,0.30)	0.10 (-0.06,0.27)	US+UE	-0.02 (-0.11,0.07)	-0.07 (-0.16,0.02)
0.16 (0.01,0.31)	0.12 (-0.03,0.28)	0.02 (-0.07,0.11)	UE	-0.05 (-0.12,0.03)
0.21 (0.07,0.35)	0.17 (0.03,0.31)	0.07 (-0.02,0.16)	0.05 (-0.03,0.12)	US
**C. League table on PPV**				
US+CEUS	CEUS	US+UE	UE	US
US+CEUS	-0.07 (-0.18,0.04)	-0.18 (-0.31, -0.05)	-0.20 (-0.32, -0.08)	-0.22 (-0.33, -0.11)
0.07 (-0.04,0.18)	CEUS	-0.11 (-0.24,0.02)	-0.13 (-0.25, -0.00)	-0.15 (-0.26, -0.04)
0.18 (0.05,0.31)	0.11 (-0.02,0.24)	US+UE	-0.02 (-0.09,0.05)	-0.04 (-0.11,0.03)
0.20 (0.08,0.32)	0.13 (0.00,0.25)	0.02 (-0.05,0.09)	UE	-0.02 (-0.08,0.04)
0.22 (0.11,0.33)	0.15 (0.04,0.26)	0.04 (-0.03,0.11)	0.02 (-0.04,0.08)	US
**D. League table on NPV**				
US+CEUS	US+UE	UE	CEUS	US
US+CEUS	-0.02 (-0.13,0.09)	-0.06 (-0.16,0.04)	-0.09 (-0.19, -0.00)	-0.10 (-0.19, -0.01)
0.02 (-0.09,0.13)	US+UE	-0.04 (-0.11,0.02)	-0.07 (-0.18,0.04)	-0.08 (-0.14, -0.02)
0.06 (-0.04,0.16)	0.04 (-0.02,0.11)	UE	-0.03 (-0.13,0.07)	-0.03 (-0.09,0.02)
0.09 (0.00,0.19)	0.07 (-0.04,0.18)	0.03 (-0.07,0.13)	CEUS	-0.01 (-0.10,0.09)
0.10 (0.01,0.19)	0.08 (0.02,0.14)	0.03 (-0.02,0.09)	0.01 (-0.09,0.10)	US
**E. League table on ACC**				
US+CEUS	CEUS	US+UE	UE	US
US+CEUS	-0.08 (-0.16, -0.00)	-0.11 (-0.21, -0.01)	-0.14 (-0.23, -0.05)	-0.16 (-0.24,-0.08)
0.08 (0.00,0.16)	CEUS	-0.03 (-0.13,0.07)	-0.06 (-0.15,0.04)	-0.08 (-0.16,0.00)
0.11 (0.01,0.21)	0.03 (-0.07,0.13)	US+UE	-0.03 (-0.08,0.03)	-0.05 (-0.11,0.00)
0.14 (0.05,0.23)	0.06 (-0.04,0.15)	0.03 (-0.03,0.08)	UE	-0.02 (-0.07,0.02)
0.16 (0.08,0.24)	0.08 (-0.00,0.16)	0.05 (-0.00,0.11)	0.02 (-0.02,0.07)	US

The values in red have been expounded in the *Results* section.

#### 3.3.2 SPE

**Figure 5:**
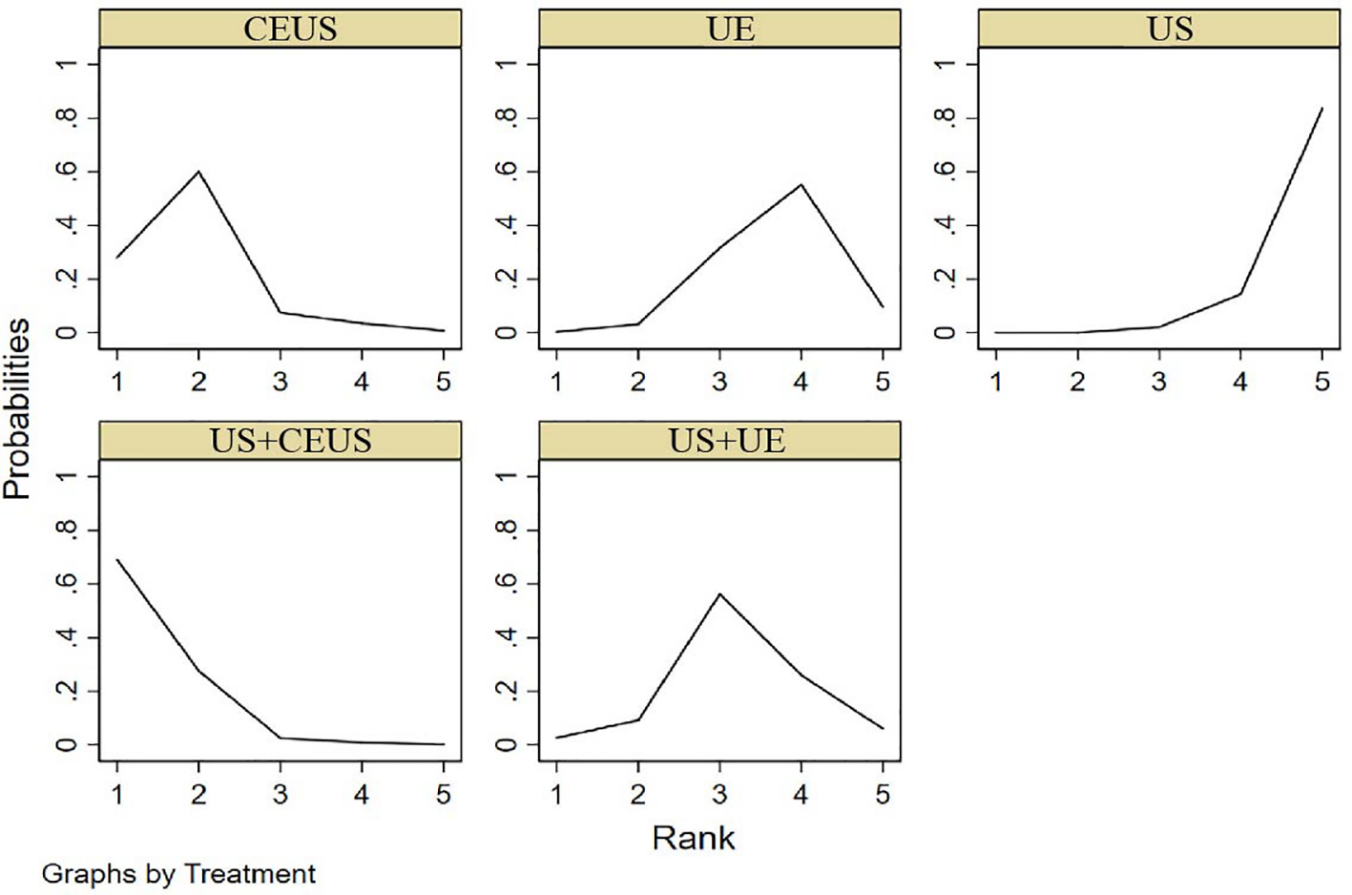
SUCRA plot for SPE.

NMA showed that US+CEUS [MD=0.16, 95% CI (0.01, 0.31)] was superior to the control group (UE) in diagnosing of SPE in axillary lymph node metastasis in breast cancer. US+CEUS [MD=0.21, 95%CI (0.07, 0.35)] and CEUS [MD=0.17, 95%CI (0.03, 0.31)] were superior to the control group (US) in diagnosing of SPE in axillary lymph node metastasis in breast cancer (**Table 4B**). US+CEUS ranked first in SPE for axillary lymph node metastasis of breast cancer in different methods(SUCRA: 90.8% as shown in **Table 5**) (**Figure 5**).

**Table 5:** SUCRA values of preoperative detection of axillary lymph node metastases in breast cancer patients by 5 different ultrasonic diagnostic methods.

Method	SEN	SPE	PPV	NPV	ACC
US C	20.5	.4.7	8.7	13.9	5.7
UE B	49.1	32.7	28.1	44.9	27.7
CEUS A	15.8	78.1	75.7	24.1	64.8
US+UE E	77.2	43.8	40.1	79.5	22.7
US+CEUS D	87.4	90.8	97.3	87.6	99.0

SEN, Sensitivity; SPE, Specificity; PPV, Positive predictive value; NPV, Negative predictive value; ACC, Accuracy.

#### 3.3.3 PPV

**Figure 6:**
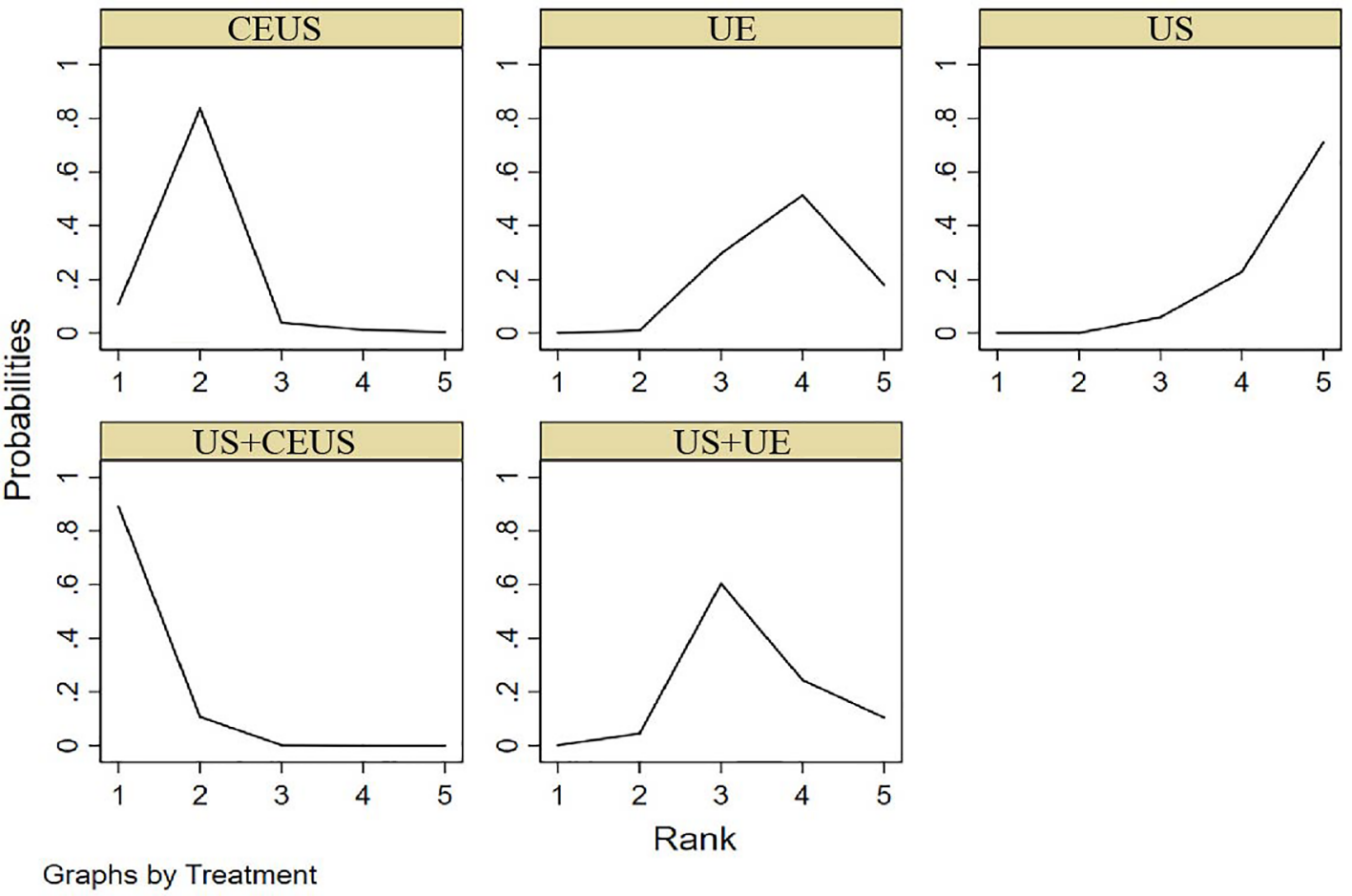
SUCRA plot for PPV.

NMA showed that US+CEUS [MD=0.18, 95%CI (0.05, 0.31)] was superior to the control group (US+UE) in diagnosing of PPV in axillary lymph node metastasis of breast cancer. US+CEUS [MD=0.20, 95%CI (0.08, 0.34)] was better than control group (UE) in diagnosing of PPV in axillary lymph node metastasis of breast cancer. US+CEUS [MD=0.22, 95%CI (0.11, 0.33)] and CEUS [MD=0.15, 95%CI (0.04, 0.26)] were superior to the control group (US) in the diagnosing of PPV in axillary lymph node metastasis of breast cancer (**Table 4C**). US+CEUS ranked first in PPV for axillary lymph node metastasis of breast cancer in different methods (SUCRA: 97.3% as shown in **Table 5**) (**Figure 6**).

#### 3.3.4 NPV

**Figure 7:**
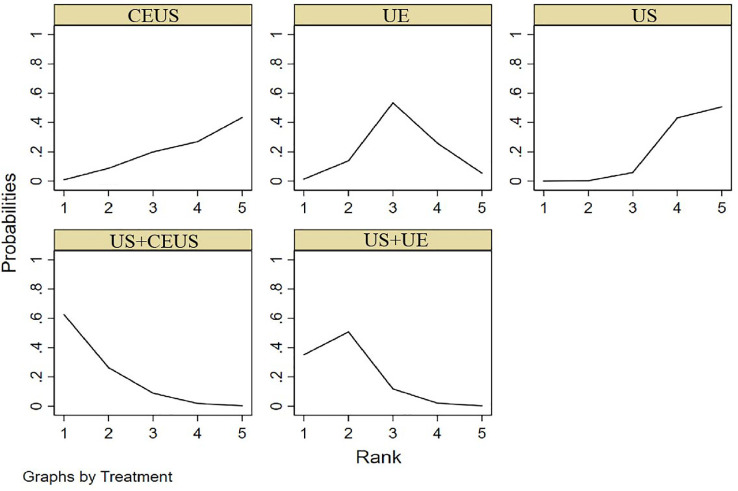
SUCRA plot for NPV.

NMA showed that US+CEUS [MD=0.10, 95%CI (0.01, 0.19)] and US+UE [MD=0.08, 95%CI (0.02, 0.14)] were superior to the control group (US+UE) in diagnosing of NPV in axillary lymph node metastasis of breast cancer (**Table 4D**). US+CEUS ranked first in NPV for axillary lymph node metastasis of breast cancer in different methods (SUCRA:87.6% as shown in **Table 5**) (**Figure 7**).

#### 3.3.5 ACC

**Figure 8:**
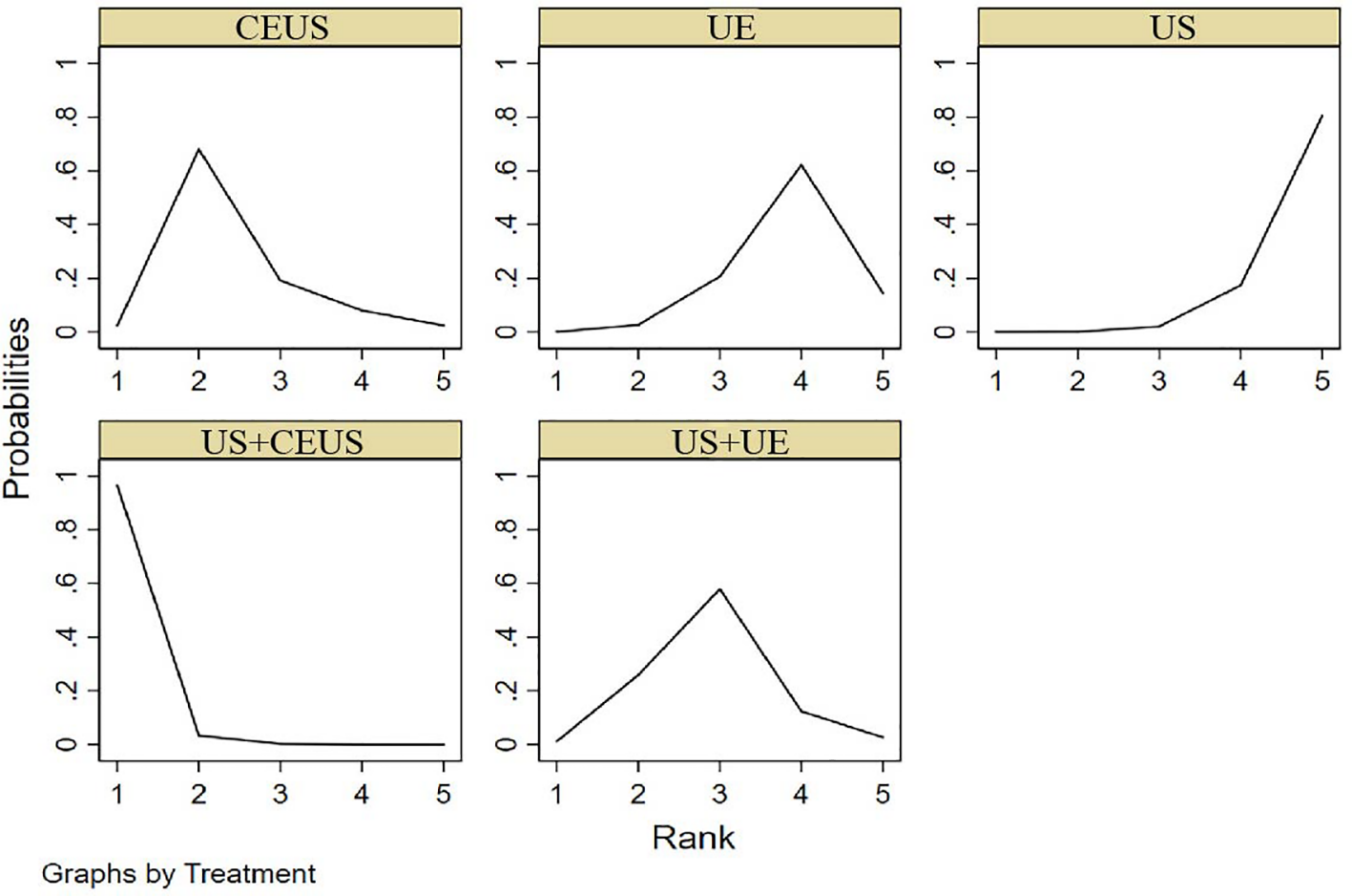
SUCRA plot for ACC.

NMA showed that US+CEUS was superior to the control group in diagnosing of ACC in axillary lymph node metastasis of breast cancer (US+UE, UE, US) (**Table 4E**). US+CEUS ranked first in ACC for axillary lymph node metastasis of breast cancer in different methods(SUCRA:99.0% as shown in **Table 5**) (**Figure 8**).

## 4 Discussion

Early identification of axillary lymph node metastasis in breast cancer is crucial for the prognosis and treatment of breast cancer patients, and SLNB is a necessary means to detect whether breast cancer has lymph node metastasis (32). However, SLNB usually carries a risk of acute or long-term complications including nerve damage, lymphedema, and wound infection etc (33). Therefore, accurate prediction of axillary lymph node metastasis in breast cancer by non-invasive diagnosis is an urgent problem to be solved. This study evaluated the diagnostic performance of US, UE, CEUS, US+UE, and US+CEUS of axillary lymph node metastasis in breast cancer patients with in detail from five aspects: SEN, SPE, PPV, NPV, and ACC. This is the first systematic review and NMA of non-invasive imaging modalities of ultrasound diagnostic methods in patients with pathologically confirmed breast cancer with axillary lymph node metastases. A total of 22 articles were included in this study, with a total of 7776 patients (range, 42-313), The combined ultrasound method was significantly better than the single ultrasound method in the diagnosis of axillary lymph node metastasis in breast cancer. Compared with other diagnostic methods, US+CEUS showed obvious advantages in predicting axillary lymph node metastasis in breast cancer in all aspects. The SUCRA values showed that CEUS had higher SEN and higher accuracy than US and UE alone in a single diagnostic method. Our analysis showed that US+CEUS could be an effective non-invasive diagnostic method for clinical diagnosis of axillary lymph node metastasis in breast cancer.

The US is considered to be a routine non-invasive diagnostic method for diagnosing axillary lymph node metastasis in breast cancer. The status of axillary lymph node s is usually assessed by blood flow, size, and shape. However, US diagnosis usually relies on the doctor’s own experience and skills, and there may be a higher misdiagnosis rate, and its SEN and SPE are quite different (27). The SPE and SEN of this diagnostic method in this study were 70% and 86%, respectively, similar to the results of Qing.Z et al. (28). UE is widely used in the diagnosis of superficial organs and lymph node metastases. Wang J et al. (24) believed that traditional two-dimensional ultrasound technology is not ideal for the differential diagnosis of breast cancer axillary lymph node metastases, while UE can accurately reflect tissue stiffness. Thus, the types of breast cancer axillary lymph node metastasis can be identified semi-quantitatively. We analyzed the 12 included articles and found that the SEN and SPE of UE for breast cancer axillary lymph node metastasis were 83% and86%, respectively, which were consistent with the results of Choi J.J et al (15).

The morphology of lymph nodes and blood flow distribution are studied using conventional ultrasound, although it is difficult to identify small infiltrative foci that do not result in morphological changes in lymph nodes; Doppler ultrasound is unable to detect anterior lymph nodes because of its low signal-to-noise ratio, inability to see microvessels, and difficulty displaying tissue perfusion. The examination of abdominopelvic and superficial organ lesions as well as the detection of SLN in breast cancer have all benefited from the widespread use of CEUS, a novel technology for the dynamic assessment of tissue perfusion utilizing ultrasonic contrast agent (UCA). It has been commonly used for the diagnosis of benign and malignant breast cancer and the assessment of axillary lymph node metastasis. Ultrasonography under enhanced conditions can reveal some of the new and immature tissues around the tumor, and the boundary and internal blood flow of the primary breast cancer are more clearly shown compared to conventional ultrasound. It is mainly by injecting a contrast agent into the patient’s body to enhance the outline of the axillary lymph node according to the concentration of the contrast agent in the patient’s axillary lymph nodes. compared with normal lymph nodes, metastatic lymph nodes showed longer duration of enhancement as well as higher imaging intensities. CEUS has been shown to be more accurate than other ultrasound methods in previous studies, and our study showed the same results with a SEN and SPE of 82% and 88%, respectively.

Most of the current clinical prediction models of axillary lymph node metastasis of breast cancer are based on clinicopathological characteristics such as age, tumor size, and histological grade. However, these clinicopathological features are usually acquired intraoperatively or postoperatively, and the diagnostic performance of single diagnostic imaging is not ideal. Therefore, we analyzed combined diagnostic methods, such as US+CEUS, and US+UE. Compared with previous studies, the combined diagnostic method was significantly higher than the single diagnostic method in terms of diagnostic performance, especially the US+CEUS combined diagnostic method showed satisfactory predictive results in terms of SEN and SPE, the mean reason is that conventional ultrasound must first locate lymph nodes in order to distinguish between benign and malignant ones; however, some lymph nodes are challenging to distinguish from nearby tissues and are frequently missed. However, some lymph nodes are hard to spot in the tissues around them and are frequently missed. By using enhanced microbubbles to detect these occult lymph nodes, CEUS can aid in their detection. It can also correct some lymph nodes that conventional ultrasonography incorrectly labeled as benign due to minor metastases. Traditional ultrasonography misdiagnoses lymph nodes as benign because of minor metastases. The combined diagnosis of the two can offer a thorough assessment of the lymph nodes’ size, shape, internal structure, and lymphatic drainage, and evaluation of the internal anatomy, lymphatic drainage, size, morphology, and diagnostic value of axillary lymph nodes. There were still some limitations in the study. First, this study needs to include kinds of literature containing two or more diagnostic methods. However, it is found that the number of such articles is limited through search, resulting in an uneven number of studies on each diagnostic method. Second, some of the results of this study may have an impact on the results of the study due to differences in the number of patients between studies. Third, due to the differences in the experience level of the radiologist in the diagnosis of diseases, there are potential differences in the studies. In view of the above deficiencies, it is suggested that readers should reasonably refer to and select the diagnostic method of this study according to clinical practice and actual results.

In conclusion, the analysis of this study showed that single US, UE, and CEUS have limited diagnostic performance in diagnosing axillary lymph nodes metastases in breast cancer. Compared with single ultrasound imaging, US + CEUS have highest diagnostic performance of axillary lymph nodes metastasis in breast cancer in the combined diagnosis, which can provide a reliable basis for breast cancer axillary lymph nodes metastasis, However, due to the lack of literature, more prospective studies are still needed to confirm this conclusion.

## Data availability statement

The original contributions presented in the study are included in the article/supplementary material. Further inquiries can be directed to the corresponding authors.

## Author contributions

Study concept and design: S-RW, Q-LL. Acquisition of data: Q-LL, MC, S-RW, TZ. Analysis and interpretation of data: S-RW, TZ. Drafting of the manuscript: TZ, P-SZ. Critical revision of the manuscript for important intellectual content: JL. Approval of the final manuscript: JL, S-RW. Study supervision: JL. All authors contributed to the article and approved the submitted version.
